# CMR-Based Risk Stratification of Sudden Cardiac Death and Use of Implantable Cardioverter–Defibrillator in Non-Ischemic Cardiomyopathy

**DOI:** 10.3390/ijms22137115

**Published:** 2021-07-01

**Authors:** Laura Keil, Céleste Chevalier, Paulus Kirchhof, Stefan Blankenberg, Gunnar Lund, Kai Müllerleile, Christina Magnussen

**Affiliations:** 1 Clinic for Cardiology, University Heart and Vascular Center Hamburg Eppendorf, 20246 Hamburg, Germany; p.kirchhof@uke.de (P.K.); s.blankenberg@uke.de (S.B.); kamuellerleile@uke.de (K.M.); c.magnussen@uke.de (C.M.); 2 Deutsches Zentrum für Herz-Kreislauf-Forschung e.V. (German Center for Cardiovascular Research), Partner Site Hamburg/Kiel/Lübeck, 20246 Hamburg, Germany; 3 Department of Diagnostic and Interventional Radiology, University Hospital Hamburg Eppendorf, 20251 Hamburg, Germany; g.lund@uke.de

**Keywords:** sudden cardiac death, non-ischemic cardiomyopathy, dilated cardiomyopathy, risk stratification, cardiovascular magnetic resonance imaging

## Abstract

Non-ischemic cardiomyopathy (NICM) is one of the most important entities for arrhythmias and sudden cardiac death (SCD). Previous studies suggest a lower benefit of implantable cardioverter–defibrillator (ICD) therapy in patients with NICM as compared to ischemic cardiomyopathy (ICM). Nevertheless, current guidelines do not differentiate between the two subgroups in recommending ICD implantation. Hence, risk stratification is required to determine the subgroup of patients with NICM who will likely benefit from ICD therapy. Various predictors have been proposed, among others genetic mutations, left-ventricular ejection fraction (LVEF), left-ventricular end-diastolic volume (LVEDD), and T-wave alternans (TWA). In addition to these parameters, cardiovascular magnetic resonance imaging (CMR) has the potential to further improve risk stratification. CMR allows the comprehensive analysis of cardiac function and myocardial tissue composition. A range of CMR parameters have been associated with SCD. Applicable examples include late gadolinium enhancement (LGE), T1 relaxation times, and myocardial strain. This review evaluates the epidemiological aspects of SCD in NICM, the role of CMR for risk stratification, and resulting indications for ICD implantation.

## 1. Sudden Cardiac Death in Non-Ischemic Cardiomyopathy: General Aspects

Sudden cardiac death (SCD) is one of the major causes of death accounting for approximately 15–20% of all deaths worldwide [1]. Despite decreasing cardiovascular mortality over the past 20 years, survival rates for out-of-hospital cardiac arrest remains as poor as 10% [2,3]. It is commonly assumed that SCD results from lethal arrhythmias. In this context, non-ischemic cardiomyopathy (NICM) is considered the second most important entity for arrhythmias and SCD next to ischemic heart disease, as approximately 30–40% of patients present with non-ischemic heart failure (HF) [4]. NICM encompasses above all dilated cardiomyopathy (DCM), hypertrophic cardiomyopathy (HCM), and arrhythmogenic right ventricular cardiomyopathy (ARVC) [1]. As many studies do not differentiate into NICM subtypes, this paper includes all entities, but it uses the term “DCM” or “non-ischemic dilated cardiomyopathy” (NIDCM) whenever clearly indicated in the literature.

Current guidelines recommend ICD implantation for patients with symptomatic HF (NYHA Class II–III) and left-ventricular ejection fraction (LVEF) ≤ 35% under optimal medical treatment, referring to ischemic cardiomyopathy (ICM) as well as NICM [5]. This is mainly based on the Sudden Cardiac Death in Heart Failure Trial (SCD-HeFT), which investigated all-cause mortality in patients with NICM and ICM after ICD implantation vs. amiodarone medication. The trial did not include patients with NYHA class I or LVEF ≥ 35% [6]. Conversely, the Maastricht registry showed that most patients who died from SCD had a preserved or mildly depressed left ventricular function [7]. Underlying these findings, a meta-analysis on predictors for SCD including a total of 6,088 patients found that LVEF had a sensitivity and specificity as low as 71% and 50% in this context [8]. The predictive value could be improved by adding MR parameters such as late gadolinium enhancement (LGE) location and pattern to discriminate different cardiomyopathies [9]. Furthermore, these parameters are able to provide early information for an underlying etiology and myocardial phenotypes predisposing to SCD, which a sole analysis of left ventricular function cannot provide.

On top of that, the underlying causes for NICM and ICM are completely different. ICM results from coronary artery disease (CAD) and myocardial infarction. It is commonly assumed that ventricular arrhythmias (VA) originate from post-infarction scar in ICM, i.e., fibrosis [2,3,10,11,12]. Fibrosis represents a substrate for VA [13] inducing electrical dispersion, slow impulse conduction, and nonuniform anisotropy. These mechanisms create re-entry circuits, which form the basis for arrhythmias [14,15]. Apart from re-entry mechanisms following myocardial fibrosis, primary arrhythmias may as well occur in NICM due to genetic defects [16].

On the other hand, DCM as a major representative of NICM is characterized by a dilated left ventricle and systolic dysfunction after the exclusion of CAD, hypertension, and valvular disease [17]. The underlying causes are heterogeneous, including 20–50% idiopathic, toxic, infectious, and genetic etiologies [18]. In DCM, fibrosis as a substrate for VA is thought to be less extended, less confluent, and more heterogeneous than in ICM. This was shown in studies evaluating the prevalence and characteristics of late potentials. Late potentials represent myocardium where conduction is slowed by fibrosis. The underlying molecular mechanisms for the difference in fibrosis between ICM and NICM are still unclear [19,20]. In ICM, scar tissue, i.e., fibrosis, involves the subendocardium following a coronary artery territory. In NICM, fibrosis can be located endocardially, epicardially, intramurally, or exhibit a diffuse pattern, depending on the etiology [21,22].

## 2. NICM and ICD Indication for Primary Prevention: Current Evidence

Nearly one third (30%) of patients with DCM die of SCD [23], and DCM is the most common cause for heart transplantation [24]. Sammani et al. found in a meta-analysis an annual rate for VA of 4.5% in patients with DCM [25]. On the other hand, the Defibrillator in Non-Ischemic Cardiomyopathy Treatment Evaluation (DEFINITE) and the Danish Study to Assess the Efficacy of ICDs in Patients with Non-Ischemic Systolic Heart Failure on Mortality (DANISH) trial showed no overall survival benefit in patients with NIDCM or NICM, respectively, despite halving the risk for SCD [26,27]. Similarly, the long-term follow-up of the SCD-HeFT showed no survival benefit for patients with NICM after 10 years [28].

Subsequently, it has been assumed that the varying findings result from the different standard of medical care at the conduction time of the above-mentioned trials. For instance, only the minority of patients in the initial SCD-HeFT was treated with mineralocorticoid-receptor antagonists, and both in SCD-HeFT and DEFINITE, no cardiac resynchronization therapy (CRT) was used [6,26,27].

Various investigations already demonstrated the favorable effect of CRT on cardiac reverse modeling and outcome [29,30,31]. Notably, reverse cardiac remodeling is more evident in patients with NICM as compared to ICM [32,33]. As a consequence, patients with NICM may experience a more pronounced overall survival benefit from CRT therapy irrespective of additional ICD as compared to patients with ICM.

On top of that, by optimal medical treatment alone, one third of patients with idiopathic DCM exhibits reverse cardiac remodeling. As a consequence, cardiovascular mortality can be reduced by approximately 25% [34]. In accordance, current ESC HF guidelines recommend ICD implantation only after persistent LV dysfunction after 3 months of optimal medical treatment [5]. Merlo et al. found that although 15% of patients showed a recovery of LV function after a mean follow-up of 19 months, 33% of these patients suffered again from a deterioration of LV function in the long-term follow-up. Notably, no predictors apart from significant mitral insufficiency at baseline could be identified [35]. Irrespective of the effects of reverse remodeling, future studies should identify patients to be referred for instant ICD implantation due to irreversible risk factors for SCD.

Importantly, ICD only protects from lethal arrhythmias, but not from other causes of cardiac death, e.g., acute HF, which is frequent in DCM patients [18]. Thus, ICD does not reduce overall mortality, as shown in the DANISH trial. This is also reflected by the overall low rate of adequate ICD therapies within DANISH, SCD-HeFT and DEFINITE [6,26,27]. In the group treated with ICD in the DANISH trial, only 4.3% suffered from SCD, while 9.5% died from other cardiovascular death [27].

Finally, adverse effects of ICD treatment have to be taken into account. After ICD-implantation, approximately 10% of patients experience at least one complication within the first 6 months [36]. Moreover, 11–23% of patients receive inadequate ICD shocks, which are mainly related to atrial fibrillation or supraventricular tachycardia, resulting in an impaired quality of life [37,38,39,40] and in a doubled mortality risk [38]. Affected patients tended to be younger and suffered more often from NICM [18,41].

That raises the question of how to determine the subgroup of NICM patients most susceptible for arrhythmias and SCD who therefore might benefit the most from ICD implantation.

## 3. Risk Stratification by Clinical Parameters, Genetics, and Non-MR Imaging

### 3.1. Clinical Risk Predictors and Current Risk Scores

Various possible predictors have been discussed in the past, to name but a few: Nonsustained ventricular tachycardia (NSVT), TWA, NYHA class, and QRS duration. The next section will give a short overview of the selected criteria and discuss the available risk scores. We chose especially NSVTs and TWA as representatives of electrical markers because of their clinical accessibility and their presence in recent meta-analyses [8,25,42,43]. However, the predictive value of non-invasive variables is limited due to contradictory study findings and low discriminate yield for high-risk vs. low-risk patients [5,43].

NSVTs occur in 40–60% of patients with idiopathic DCM [21] and have repeatedly been associated with malignant arrhythmias [25]. Gigli et al. found that in DCM with only mild LV dilatation, NSVTs were an independent predictor for death and heart transplantation [44]. Similarly, DCM patients on optimal medical treatment and an LVEF >35% and NSVTs were shown to have a higher risk of malignant VA than those without [45]. Recently, in ICD-implanted DCM patients, rapid-rate NSVTs were associated with subsequent VA [46]. Nevertheless, the study findings are contradictory, and some authors suggest NSVTs rather as a marker for progredient HF than for arrhythmic events [47,48].

TWA has also been discussed as valid risk predictor. It describes the morphologic change of the T-wave in every heartbeat [25]. However, inconsistent results and the lack of a large dataset are drawbacks to possible risk stratification [42,47,48,49].

In a recent meta-analysis, the authors analyzed possible predictors for VTs in DCM. They found a significant association for hypertension, genetic mutations (LMNA, PLN, and FLNC), TWA, and the presence of LGE. Interestingly, family history of SCD and NYHA class did not fulfill statistical significance [25]. Another group suggested an assessment at different time points, finding different predictors in the course of the disease. For example, QRS duration and mitral regurgitation were associated with arrhythmic risk at baseline, whereas left atrial area and indexed LV end-diastolic volume were predictors at later stages [50]. This approach may be interesting considering the varying course of DCM, although it may not be very easily applicable in clinical routine.

Lately, Younis et al. introduced two scores for VT/ventricular fibrillation (VF) vs. non-arrhythmic mortality based on all four MADIT trials and described a predictive value for sex, age, prior atrial arrhythmia, LVEF, systolic blood pressure, prior myocardial infarction, and heart rate. In accordance with the above-mentioned studies, older patients or patients with higher comorbidity burden (diabetes mellitus, higher NYHA class, higher body mass index) were less likely to benefit from ICD implantation [51]. The score was validated in a cohort with 49% NICM patients and can be easily used in the clinical routine via a dichotomic online tool [51]. The fact that the score includes both ICM and NICM holds certain limitations. As stated above, NICM represents a heterogenic group with distinct characteristics and with different risk factors compared to patients with ICM. For instance, NICM patients are more often younger and female than patients with ICM. Therefore, future risk scores should focus on NICM or, even more specifically, on DCM alone. Moreover, CMR findings or genetics were not incorporated [52], which might add further prognostic information.

### 3.2. Genetics and Their Additional Value for Risk Stratification

In about 30 to 50% of DCM patients, a positive family history can be found [18,41]. Today, 56 genes are commonly included in clinical testing [53]. There is growing evidence for using genetic testing in clinical routine and including genetics in individual risk stratification [23,54]. Above all, LMNA, FLNC, PLN, and RNA-binding motif protein 20 (RBM20) are associated with a higher risk for SCD in patients with DCM [41,54].

LMNA encodes for two intermediate filament proteins, lamin A and C [55]. These proteins form the meshwork underneath the inner nuclear envelope called the nuclear lamina [56,57] and play a major role for the architecture, integrity, and metabolism of the nucleus [56]. LMNA mutations can be found in up to 8% of patients with DCM [58,59] and are associated with arrhythmias, including atrial fibrillation and ventricular tachycardias (VTs) as well as conduction disorders such as atrioventricular node dysfunction and musculoskeletal disorders [58,60,61,62]. Carriers have a significantly lower survival rate than non-carriers [58] with a mean survival of 50 years [63]. SCD has been found to be especially high in this group (31 to 46%) [62,63]. Rijsingen et al. found amongst others male sex, NSVTs, and an ejection fraction of <45% as independent risk predictors for SCD, which are also adopted in the ESC guidelines for the prevention of sudden cardiac death [5,63]. In addition, non-missense mutations may impose an increased risk [64] and are mentioned in the guidelines [5]. Recently, a more accurate risk score for VA also including AV block has been proposed and validated [65]. Of note, pacemakers may not prevent SCD in this group [62,64]. In conclusion, LMNA mutation carriers should be closely monitored for the above-mentioned characteristics and may receive an ICD earlier than other DCM patients. It should be considered to implant an ICD rather than a pacemaker if necessary [66].

FLNC is the underlying gene for the protein filamin C. Filamin C is actin binding and is thought to play an essential role in the integrity and signal transduction of cell–cell connections, in the Z-disc and between sarcomeres and the cell membrane [55,67]. A truncating mutation was found in patients with DCM and was associated with LV dilatation, dysfunction, and fibrosis. Moreover, carriers are prone to VA and SCD [55,68]. As a result, a recent expert consensus recommends a primary prophylactic ICD implantation in patients with a truncating FLNC mutation and an LVEF < 45% [66].

RBM20 is involved in the post-transcriptional splicing of various proteins in cardiomyocytes, most notably titin and proteins regulating calcium homeostasis [69,70,71]. Variants in its underlying gene have been found to be associated with early onset DCM, VA, and SCD [71,72,73]. Recently, van den Hoogenhof et al. found a possible treatment option for this subset of patients in using the calcium channel blocker Verapamil [71]. Due to its high penetrance and proarrhythmic clinical manifestation, the authors suggest close monitoring of mutation carriers [72,73].

The protein phospholamban with its underlying gene PLN also plays a role in the calcium homeostasis [74]. Phospholamban inhibits the Ca^2+^-ATPase of the sarcoplasmatic reticulum, and its phosphorylation leads to muscle relaxation [75]. When mutated, the inhibitory effect is nonreversible [70]. Mutation carriers are prone to malignant disease progression, VA, cardiac fibrosis, and SCD [76,77]. It represents a founder mutation in the Netherlands, where it has been discovered in 15% of DCM patients [77], while the prevalence has been shown to be <1% in HF patients of another population [78].

### 3.3. Non-MR Imaging and Risk Prediction

#### 3.3.1. Echocardiography

Global longitudinal strain (GLS) provided by speckle-tracking echocardiography (STE) is an evolving method for the evaluation of LVEF [79,80]. Nikoo et al. demonstrated that GLS was superior in predicting VAs as compared to LVEF in a study population of 70 patients with ICM and DCM: by using a cut-off value of −10%, GLS had a specificity of 90% and sensitivity of 72.2% [80]. This is in line with an earlier study of 308 patients with chronic HF due to ICM and NICM that showed a sensitivity of 73% and specificity of 61% in predicting VA events. When combined with LVEF, the prognostic value could be increased even further [81]. More recently, Tröbs et al. showed an association of GLS with cardiac death independent of cardiac function and NYHA class in a cohort of 2186 patients with chronic HF [82]. However, few studies investigated the prognostic value of impaired GLS on arrhythmic events of patients with NICM, and randomized trials with a high number of patients and focus on NICM or, even more specific, DCM alone are lacking.

When compared to CMR, the advantages of echocardiography remain the cost-effectiveness, higher availability, and the usability irrespective of renal function or cardiac devices affecting image quality [83,84]. However, inter-observer variability was lower in CMR [83]. Nevertheless, a good correlation between CMR-FT and STE-derived GLS was shown [83,85,86].

#### 3.3.2. Single-Photon Emission Computed Tomography (SPECT)

Impaired sympathetic innervation and activity result in heterogeneous electrical conductance, act as arrhythmic substrate, and are linked to increased risk of SCD [87,88,89]. In this context, the norepinephrine analogue meta-iodbenzylguanidine radiolabeled with iodine-123 (123I-mIBG) has been used to measure neuronal integrity with reference to the heart/mediastinum (H/M) uptake ratio [90,91]. In a prospective cohort of 961 HF patients, Jacobson et al. found a higher cardiac mortality and arrhythmic risk in patients with a H/M ratio <1.60 [90]. In contrast to these findings, De Vincentis et al. failed to show an independent association between the H/M ratio and arrhythmic events in a population of 170 patients with chronic ICM or NICM [92]. In summary, current data using SPECT parameters for risk prediction are limited and controversial, which is possibly due to low accessibility, expertise, and high costs [88].

## 4. Risk Stratification by Cardiovascular MR Imaging

So far, a variety of CMR-derived parameters have been evaluated for their usefulness in risk assessment for SCD in NICM patients. An overview of these parameters including limitations are provided in Table 1.

### 4.1. Late Gadolinium Enhancement

#### 4.1.1. General Aspects

LGE imaging is an accurate and commonly applied technique for the identification of focal myocardial fibrosis and thus can visualize and quantify substrate for VA [13]. LGE results from regional abnormalities in myocardial extracellular volume due to myocardial injury, e.g., myocardial necrosis, edema, and scar tissue [93]. LGE can be characterized by its overall extension, its location within the LV, and its pattern [94]. In this review, the term “LGE” will be used to refer to focal myocardial fibrosis only.

#### 4.1.2. Presence of LGE and Association to VA

In NICM, the prognostic value of LGE for adverse cardiovascular outcomes including SCD was shown in multiple studies, including two meta-analyses [10,95]. The first meta-analysis demonstrated that NICM patients with LGE had a higher annualized event rate for a combined outcome of SCD, aborted SCD, and appropriate ICD therapy compared with patients without LGE (6.0% versus 1.2%; *p* < 0.001) [95]. The second meta-analysis revealed a lower OR for arrhythmic events of 5.05 (95% CI: 2.73 to 9.36) in studies only comprising ICM patients compared with 6.27 (95% CI: 4.15 to 9.47) in studies on NICM patients [10].

In addition, LGE was reported to be a strong independent predictor for VA and SCD after adjusting for other clinical or functional prognostic parameters [96,97,98,99,100,101]. One study analyzed NICM patients referred for primary ICD implantation. Patients with LGE had a composite outcome event including non-sudden and sudden cardiac death, ICD discharge, or hospital admission due to HF in 44% compared with 8% in patients without LGE. After controlling for EF, LV mass, LV volume, or NYHA class, LGE-positive patients still had an 8-fold higher risk for the composite endpoint [101]. The presence of LGE not only predicted the composite endpoint but also ICD firings or SCD alone. However, in a multivariable analysis of Müller at al., the presence of LGE was not an independent predictor of outcome in NICM patients. Only LVEF ≤ 40% and elevated Troponin I ≥0.03 µg/l independently predicted a composite of all-cause mortality, heart transplantation, aborted SCD, sustained VT, or hospitalization due to decompensated HF [102].

For NIDCM in particular, the LGE-related risk for VA and SCD was also described by two meta-analyses [97,98], several prospective studies [96,99,103,104,105,106,107,108], and also several retrospective cohorts [109,110,111,112,113]. The meta-analysis by Becker et al. showed that LGE-positive patients had an OR of 4.52 (CI 3.41–5.99) for a combined endpoint of VA [98]. A review by Aljaroudi et al. [9] looked at six studies [99,101,114,115,116,117,118] with different endpoints including sustained VT, inducible VT, ICD therapy, SCD, or hospitalization for HF. In all studies, myocardial scar by LGE represented an independent predictor of adverse outcome. Studies focusing on a specific subset of DCM, such as patients with lamin A/C mutations or muscular dystrophies, could also confirm the prognostic relevance of LGE in the assessment of VA and SCD [119,120].

A severely reduced EF is the central parameter in current guidelines for risk stratification of SCD. However, evaluation of LGE as a risk predictor has not only yielded interesting results in patients with severely reduced EF but also in patients with mild to moderate LV dysfunction and even preserved EF. In DCM patients with an LVEF > 40%, LGE predicted the composite endpoint of SCD and aborted SCD (HR, 9.3; 95% CI, 3.9–22.3; *p* < 0.0001) [96]. A meta-analysis of Di Marco et al. examined twenty-nine studies, with a wide spectrum of DCM and a mean EF between 20% and 43%. The association between LGE and an arrhythmic endpoint was significant in studies with a mean EF below 35% as well as in those with an EF above 35%. However, the association was stronger in populations with a mean EF > 35% (EF > 35%, OR: 5.2, 95% CI: 3.4 to 7.9; EF < 35%, OR: 4.2, 95% CI: 2.4 to 7.2) [97]. On the contrary, others showed a doubling in risk for an arrhythmic event in case of detected LGE and an EF < 30% compared to an EF > 30% [10]. In some studies, LGE predicted adverse events, although LVEF did not. In a group of NICM patients, adding LVEF to a multivariate prediction model with clinical data for a combined cardiovascular endpoint of cardiac death, onset of chronic HF, and aborted SCD did not improve the prediction of outcome. Yet, including the presence or extent of LGE in the model significantly supported outcome prediction [100]. Likewise, Neilan et al. could demonstrate that while LVEF was not predictive, the presence and extent of LGE was the strongest predictor of recurrent events in survivors of SCD [121]. A recent study in NICM also showed that LGE was strongly related to SCD, while there was no significant association between LVEF ≤ 35% and the risk for SCD [122]. Another study in NICM patients with a LVEF < 35% reported that implantation of ICD led to decreased mortality only in patients with detectable LGE (without LGE: HR = 1.22, CI: 0.53–2.78, *p* = 0.64 vs. with LGE: HR = 0.45, 95% CI: 0.26–0.77, *p* = 0.003) [123].

Electrophysiological studies using CMR-guided substrate ablation further support the inclusion of LGE into risk stratification. They indicate that LGE comes close to what is identified as arrhythmic substrate. LGE is displayed in the form of color-coded PSI maps which are obtained from high spatial resolution CMR images. They can be added to the navigation system to assist VT ablation and have a reasonably high correlation with the electroanatomic maps (EAMs). In a study with a mixed cohort of NICM and ICM patients, there was lower VT inducibility and fewer VT recurrences with CMR-aided substrate ablation than in the control group [124]. The authors considered the technique to be particularly promising in NICM [125]. A prior study solely investigating NICM patients could make similar observations [126].

Overall, there is substantial evidence for an association of LGE and risk for VA in NICM, which is also independent from traditional risk factors such as EF.

#### 4.1.3. Extent of LGE and Association to VA

Methods to assess LGE comprise visual analysis, the evaluation of signal intensity values of 2 to 6 standard deviations (SD) above the intensity of remote myocardium [127,128,129], the evaluation of signal intensity >50% of the maximal signal intensity within the enhanced area (= full-width at half maximum) [130], or the evaluation of signal intensity above peak remote myocardium [131]. In ICM, the so-called “gray zone” defines a region at the periphery of myocardial infarction, where the viable myocardium is intertwined with fibrosis and therefore has an intermediate signal intensity. This zone is suspected to be particularly vulnerable for generating VA [12,131,132]. In NICM, no “gray zone” equivalent has been described so far. The literature on the relation of LGE extent and manifestation of VA contains divergent results [97,133]. Although some studies indicate a clear association between the amount of scar and arrhythmic risk, others do not. In NICM, studies confirming a relationship showed LGE extent to be associated with SCD [104], the occurrence of VT [134], and a composite endpoint of cardiovascular death and VA [108]. Some studies additionally demonstrated that LGE extent is more predictive than the mere LGE presence alone. The extent of total scar in the form of fibrosis was the most important predictive parameter in a mixed group of ICM and NICM patients [114] and in groups of NICM patients only [99,117]. A recent study, using no arrhythmia-specific endpoint but a primary outcome of all-cause mortality also found that LGE extent showed stronger associations to the outcome compared to LGE presence alone [135].

Studies have also assessed arrhythmic risk with respect to specific cut-offs in LGE extent. In a study population undergoing evaluation of ICD implantation, with half of patients suffering from ischemic heart disease, an LV scar size >5% was found to be the strongest predictor of the primary endpoint of death of VT or appropriate ICD discharge for VT. Patients with an LVEF > 30% and a scar with >5% of LV mass were determined to have a high risk for VA compared to patients with an LVEF < 30%. In contrast, patients with an LVEF < 30% and minimal or no scar had a low risk for VA, similar to those with an EF > 30% [114]. However, although LGE above 5% of LV mass was accompanied with significant additional risk for VA, the association reached a plateau at higher levels of LGE extent [114].

In a population with NICM, the percentage of LGE that predicted a primary outcome of death and hospitalization was 4.8% [99]. Lehrke et al. reported a similar LGE cut-off in DCM. LGE with >4.4% of LV mass was an optimal discriminator for a composite cardiovascular endpoint of cardiac death, hospitalization for decompensated HF, or ICD firing [106]. Neilan et al. defined a slightly higher threshold with LGE involving >6.1% of LV myocardium [108]. However, none of these studies assessed an endpoint only comprising arrhythmic events. Focusing on a pure arrhythmic endpoint, Piers et al. defined an optimal cut-off of LGE with a mass ≥7.2 g to predict monomorphic VT in NICM patients who underwent ICD implantation (AUC 0.84). However, LGE extent did not predict occurrence of polymorphic VT or VF [111].

In addition to defining specific cut-offs, studies investigated a possible association of the transmurality of scars with adverse outcomes. In 26 NICM patients, a scar involving 26% to 75% of wall thickness was discovered to be the most significant predictor of inducible VT [117]. In another study, risk for monomorphic VT was especially high when LGE showed 51–75% of transmurality [111].

Contrary to the above stated studies, others found only a limited value of LGE extent for prediction of SCD or VA [96,105,113,136]. For example, Halliday et al. could not relate LGE extent to a primary endpoint of aborted SCD in DCM patients. Patients with an LGE extent of up to 2.5% showed a similar HR as patients with an LGE extent >5% (HR 10.6 (95% CI, 3.9–29.4) vs. 11.8 (95% CI, 4.3–32.3)) [96]. This was also reported in further studies [105,113].

In summary, the association of LGE extent and risk for VA still remains unclear. Although some studies report an incremental risk with greater LGE extent and similar cut-offs of around 5% of LV mass, other studies do not attribute additional prediction value to the extent of LGE on top of the presence of LGE alone.

#### 4.1.4. Location and Pattern of LGE and Association to VA

**Figure 1:**
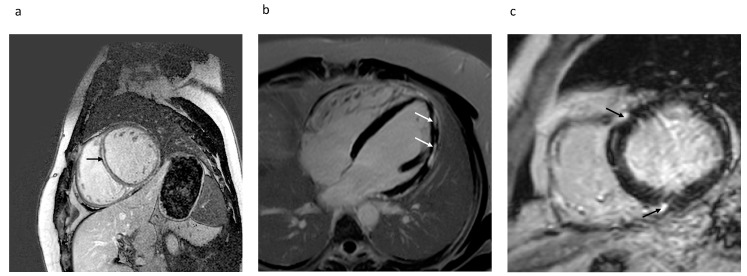
Different late gadolinium enhancement (LGE) patterns in non-ischemic cardiomyopathy (NICM). (**a**) Septal midwall LGE in short-axis view; (**b**) subepicardial patchy LGE of the lateral wall in four-chamber long-axis view; (**c**) patchy LGE at right ventricular (RV) insertion points in short-axis view.

Beyond the presence and extent of LGE, the predictive value of location and pattern of LGE has also been investigated. The most common LGE patterns in NICM are linear midwall, subepicardial, or patchy enhancement patterns not following a coronary artery territory [101]. Some patients also present an infarct-like pattern in the absence of CAD, presumably after coronary spasms or embolic events. Studies mainly differentiate between septal and free-wall location. Examples of different LGE patterns and localizations are given in Figure 1.

NICM patients with septal midwall LGE were reported to be at an increased risk for SCD [99,102,104,107,109]. Some studies associated septal midwall LGE with greater risk for SCD, exceeding the risk of other distribution patterns. For instance, Almehmadi et al. found that in patients with systolic dysfunction due to mixed etiology, septal midwall LGE was the exclusive predictor of SCD or appropriate ICD therapy among different LGE patterns [137]. Shin et al. showed that a subepicardial distribution of LGE in NICM patients was an independent predictor of a composite of major arrhythmic events [138].

In a different predictive model applied in NICM patients, coexistent septal LGE and free-wall LGE indicated a higher risk for SCD. The combination of LGE presence and location was superior to the combination of LGE extent and pattern [105]. Out of the different visually assessed LGE distributions in NICM patients, Mikami et al. also reported septal and/or RV insertions site fibrosis to strongly predict a composite endpoint of cardiac mortality or appropriate ICD discharge. Additionally, the authors found that septal LGE with approximately 3% or more of the LV mass was linked to a 9-fold higher risk of cardiac death or appropriate ICD therapy [139].

Piers et al. compared a basal vs. non-basal distribution of LGE in NICM patients who underwent ICD implantation. They were followed for the occurrence of VA [111]. Basally located LGE was a stronger predictor for monomorphic VT. VT ablation studies have also reported that substrates for monomorphic VT in NICM show a predominantly basal location [140].

Other studies have not demonstrated an association of LGE location and additional SCD risk in NICM and NIDCM [101,106,108,109,112,113,117,137]. In a study of NICM patients, LGE was strongly related to arrhythmias regardless of the segmental pattern. Multivariate analysis showed that both septal and lateral midwall LGE were associated with arrhythmias [109]. Electrophysiological studies also reported no significant relationship between the location of LGE and the occurrence or inducibility of VT (*p* = 0.60) [117,134].

In summary, current data concerning the role of LGE localization and pattern are partly contradictory, and the distribution associated with the highest risk for VA is still unclear.

#### 4.1.5. Limitations of LGE

Applying LGE as a risk predictor for the assessment of SCD holds limitations.

First, there are limitations inherent to the technique of MRI [141], such as partial volume effects, long acquisition time, costs, as well as restrictions to the use of a contrast agent in case of renal impairment, which is often concomitant to advanced NICM.

Second, the different assessment methods to define scars make an overall comparison of results difficult. However, a meta-analysis could show that significant associations between LGE and VA or SCD were preserved in studies with visual analysis of LGE and in studies with threshold-based methods [97]. Approaches to the quantification of scar extent are also heterogeneous. Extent is either defined as the sum of hyperenhanced segments [100,142,143], percentage [104,108,139], or absolute weight [98,111]. There is also no consensus on cut-offs [115,128]. These aspects further impede the comparability of studies and complicate meta-analysis. Importantly, the absence of LGE does also not insure the absence of risk for VA [101]. LGE imaging visualizes focal myocardial fibrosis but is not able to detect diffuse fibrosis. Therefore, the application of additional CMR parameters to address diffuse myocardial fibrosis is necessary.

### 4.2. T1 Mapping and Extracellular Volume

#### 4.2.1. General Aspects

The second important CMR technique for the detection of myocardial fibrosis is T1 mapping and the assessment of extracellular volume fraction (ECV). In contrast to LGE, T1 mapping and ECV imaging are able to detect and to quantify diffuse myocardial fibrosis. Briefly, T1 mapping assesses longitudinal, T1 relaxation times of myocardial tissue. Absolute T1 relaxation times are illustrated pixel-wise on T1 maps. Myocardial T1 times reflect myocardial tissue composition and e.g., can be altered due to an excess in free water and collagen, but also protein, lipid, or iron deposition. Myocardial ECV can be estimated from myocardial and blood T1 times before and after contrast agent administration as well as the hematocrit and targets the relative proportion of myocardial extracellular space [144,145]. Diffuse myocardial fibrosis results in longer native T1 relaxation times compared with normal myocardium [145,146]. Collagen deposition and consecutive expansion of extracellular space can be assessed by an increased contrast-media distribution volume with subsequently shortened post-contrast T1 relaxation times [145,147,148]. A recent meta-analysis revealed an overall favorable correlation between pre- and post- T1 mapping values, as well as ECV and histological analysis in different types of cardiac diseases [149]. Native and post-contrast T1 values provide a high diagnostic accuracy, sensitivity, and specificity in the discrimination of normal and diffusely diseased myocardium in DCM [150].

#### 4.2.2. Role in NICM

Results from ECV and T1 times analyses are especially valuable in NICM, as patients are suspected of having considerable diffuse fibrosis besides focal scarring [151], which has also been histologically confirmed [152]. Diffuse fibrosis is thought to form an essential part in the remodeling process of NICM [151]. The mechanisms of arrhythmias in diffuse fibrosis are less understood but are expected to depend on re-entry mechanisms as well [151]. T1 mapping and ECV calculation can also depict fibrosis in the absence of LGE [133,153,154]. Additionally, studies demonstrated that changes in T1 values are already present at early stages of DCM, when LVEF is only mildly reduced [155].

Abnormal T1 or ECV values were described as independent predictors for adverse clinical outcomes in various cardiomyopathies [156] and also HF with preserved ejection fraction [157,158].

In NICM, ECV was shown to independently predict cardiovascular death, HF hospitalization, and appropriate ICD shock [159]. In a larger, multicenter cohort study with 637 NICM patients, native T1 and ECV values were both strongly associated with all-cause mortality and HF [135]. The prognostic value of T1 mapping was stronger than that of LGE for the HF endpoint.

Studies investigating arrhythmic endpoints have also come to interesting results. In a mixed cohort of ICM and NICM patients undergoing ICD implantation, native T1 mapping was independently associated with an endpoint of appropriate ICD firing or documented sustained VT. Every increase of 10 ms in native T1 times increased the HR for VA by 1.06 (CI 1.01–1.11, *p* = 0.021). Remarkably, the association persisted even after correction for LGE burden. ECV showed no association, which was probably due to procedure-related limitations such as contrast kinetics and variations in hematocrit levels [160]. In another study on NICM, patients with a history of complex VA presented higher native T1 values compared to patients without any prior complex VA. Native myocardial T1 values remained associated with complex VA after controlling for LV function and LGE [151].

On the basis of prior studies, T1 mapping and ECV imaging assessment could form an important part in VA risk stratification of NICM patients. The technique could be especially interesting in patients without depicted LGE. As there is no need for contrast media application, native T1 mapping could further be useful in patients with contraindications to contrast agents.

#### 4.2.3. Limitations of T1 Mapping and Extracellular Volume

The technique of T1 mapping and ECV calculation has limitations concerning the data acquisition, the post-processing assessment method, and the interpretation of data. Mapping is known to be affected by confounding variables [161,162] such as gadolinium contrast agent dose, rate of injection, and relaxivity, and also time between T1 mapping measurement and gadolinium administration. Until now, there has been no standardization of mapping techniques [133]. Post-processing assessment methods also lack standardization. The most commonly used approach is a single-section technique at the mid-ventricle. However, this method might not adequately represent inhomogeneous fibrosis [162]. Another obstacle to the comparison of studies is that cut-offs for abnormal values are still vendor dependent. The overlap of ECV values between controls and early DCM patients, who are defined as only having mild LV dysfunction with an EF >45%, represent a further limitation to the interpretation of abnormal results [155].

Nevertheless, mapping is a valuable tool for SCD risk assessment, especially in patients with NICM. Future directives should include the standardization of data acquisition and post-processing [163].

### 4.3. Myocardial Strain

#### 4.3.1. General Aspects

Myocardial strain describes myocardial deformation [164] and is a parameter of myocardial function in addition to EF. GLS defines longitudinal shortening of the LV from base to apex. Global circumferential strain (GCS) represents LV shortening along the circular outline, and global radial strain (GRS) depicts the thinning and thickening of the LV muscle [165]. The two main techniques in the assessment of strain via CMR are MR tagging and MR feature tracking (MR-FT). CMR-FT is currently the most feasible method, as tracking can be applied to standard cine images, and no additional sequences are needed. Cut-offs for strain values vary among methods, modalities, and software [164].

#### 4.3.2. Role in NICM

Recent studies have investigated the association between myocardial fibrosis and strain abnormalities. If strain is able to accurately detect areas of fibrosis and therefore a possible substrate for arrhythmias, it could be valuable for the prediction of VA. For instance, midwall fibrosis in NICM patients was associated with reduced LV GCS, strain rate, and torsion defined by CMR-FT [166]. In end-stage DCM, histologically assessed LV myocardial fibrosis also correlated strongly with GLS. However, GLS was assessed by STE [167].

Similar to LGE and mapping, abnormal strain values were shown to be associated with prognosis in DCM patients. A study using CMR-FT found impaired GLS and mean longitudinal strain to be an independent prognostic parameter for a composite cardiac endpoint of cardiac death, heart transplantation, and aborted SCD. Strain values were shown to be superior in risk prediction compared to NYHA, EF, and LGE [103]. In a mixed cohort of non-ischemic and ischemic DCM, GLS assessed by CMR-FT showed to support risk stratification for all-cause death incremental to EF and LGE [168].

Riffel et al. assessed long axis strain (LAS) in NICM by a different method without using a deformation analysis software [169,170]. The authors measured LAS as a displacement of the mitral annulus. LAS was reported to be an independent predictor for cardiac events including aborted SCD by appropriate ICD firing. The association was described in patients with and without LGE. The authors further introduced a three-point scoring model for risk stratification, including LVEF < 35%, LAS > −10%, and the presence of LGE. Patients with three points had a significantly higher risk than those with two or less points [170].

To our knowledge, a CMR study solely focusing on an arrhythmic endpoint in NICM has not been conducted. In post-infarct patients, an MR-tagging study showed that shorter time to peak circumferential shortening strain was associated with inducible VA [171].

Overall, the results on strain in the risk assessment for VA are promising. However, the literature is still scarce, and further investigations are needed.

#### 4.3.3. Limitations of Myocardial Strain

Strain values vary depending on the assessment method and software version used [172]. Thus, method and software specific cut-off values have been implemented. However, this limits a broad and straightforward comparison of study results. Some methods have also not been properly validated. Therefore, studies have argued for a widespread validation and cross-modality as well as vendor standardization [164]. Additionally, some strain parameters, particularly radial strain values and segmental strain values, still lack reliability [164,165].

In conclusion, similar to EF where there is no available cut-off to discriminate patients at risk for sudden or non-sudden cardiac death [173], there is no single CMR parameter able to accomplish that today. CMR parameters have not been used in trials that current guidelines are based on and have not been implemented in standard risk scores [174] despite having the potential to add insightful information.

### 4.4. CMR Risk Scores

To the best of our knowledge, for NIDCM, only one CMR-based risk score has been reported so far. The ESTIMATED Risk Score [175] is the first and only algorithm that combines LGE and conventional risk factors for SCD. A total of 395 NIDCM patients with an average EF of 28.8% were recruited and followed over a 3-year period. The primary outcome was a composite of SCD events (SCD, aborted cardiac arrest, appropriate ICD therapy).

The score was developed in 295 NICM patients without prior VA. 100 patients with documented VA were used for validation. The predictive value was assessed by comparing SCD events between high-risk patients defined by the score and the validation group. The score integrated LGE extent > 14%, syncope, atrial flutter/fibrillation, NSVT, advanced AV block, and age < 20 or >50 years. The extent of LGE strongly predicted SCD risk, while an LGE extent > 14% was not detected in the low-risk group but present in nearly 100% in the high-risk group. The most important limitations of the study were the small sample size and the unspecified SCD endpoint not restricted to sudden death due to tachyarrhythmias [175]

### 4.5. CMR Parameters and their Role in Risk Stratification of SCD in HCM and RCM

Apart from DCM, there is growing evidence for MR-based risk stratification for SCD in other NICM entities, especially in HCM and restrictive cardiomyopathy (RCM).

LGE has been described in approximately 50% of HCM patients [176,177]. It can most commonly be found within hypertrophic segments and at RV insertion points in a patchy mid-myocardial pattern [178,179,180].

Several studies have shown an association of LGE with increased risk for VA or SCD [179,181,182,183,184,185], and similar to DCM, they have not only investigated SCD risk in relation to the mere presence of LGE but also in relation to LGE extent. One meta-analysis demonstrated that extensive LGE represented an independent risk marker for SCD. A 10% increase in LGE extent was linked to a 36% relative increase in risk for SCD and a total LGE extent of 20% of LV mass was associated with a nearly two-fold increase in SCD risk. [186]. Another study described a 15% increase in risk for the primary endpoint of cardiovascular death, unplanned hospital admission, sustained VT/VF, or appropriate ICD discharge for every 5% increase in fibrosis [182]. Some studies further examined specific cut-offs of LGE extent in relation to SCD risk prediction [176,187]. Chan et al. could show that an LGE extent ≥ 15% of LV mass was associated with a two-fold increase in risk for SCD. Considering LGE in addition to the traditional factors introduced by the American College of Cardiology (ACC)/American Heart Association (AHA) guidelines improved the overall risk stratification [178]. Similarly, Mentias et al. also found that LGE extent ≥ 15% of LV mass increased the risk of a composite outcome of SCD and appropriate ICD firing [180].

On the other hand, Maron et al. could not report LGE to be a risk factor for a composite primary endpoint of SCD, appropriate ICD discharge, and progressive HF symptoms of ≥1 NYHA class. Although patients with LGE reported a higher rate of adverse events compared to those without LGE, the comparison did not prove statistically significant [188].

The above-mentioned studies hold limitations due to different scanning protocols and in particular due to different LGE assessment and quantification methods [177]. As a result of this lack of a larger body of reliable evidence, guidelines up to 2019 did not implement CMR parameters as first-line criteria in risk stratification for SCD. The 2011 ACC/AHA guidelines for SCD risk stratification gave a class IIb recommendation for CMR LGE imaging in selected patients with known HCM, when SCD risk stratification was inconclusive after assessment of the conventional risk factors (a family history of SCD, history of VF or tachycardia, prior resuscitation for SCD, unexplained syncope, and maximal LV wall thickness (MWT) ≥30 mm) [176]. The HCM Risk SCD calculator also only relies on MWT, family history of SCD, NSVT, unexplained syncope in addition to left atrial diameter, maximal LVOT gradient (rest/Valsalva provoked), and age [189]. Yet, a study by Freitas et al. showed that the amount of LGE showed higher discriminative power in the identification of HCM at risk for SCD than the HCM Risk-SCD score and the ACC/AHA algorithm [190]. However, the enhanced ACC/AHA risk factor strategy of 2019 then did incorporate LGE with diffuse or extensive distribution as a major SCD risk marker in HCM. Its diffuse or extensive appearance was quantified as about 15% or more of LV mass or estimated as being extensive or diffuse through visual assessment [188]. The adapted strategy proved to be highly sensitive for predicting SCD events.

The ESC 2014 guidelines stated that although LGE imaging might be useful in predicting cardiovascular mortality, the data did not allow using LGE in standardized risk prediction for SCD [177]. In a study of Hinojar et al., the extent of LGE was an independent predictor between ESC low- and high-risk groups with nearly all of the high-risk patients showing an LGE extent > 15%, but they did not support distinguishing intermediate from high-risk patients [191].

In addition to LGE imaging, myocardial mapping and risk for VA have also been studied in HCM. One study found heightened T2 times consistent with myocardial edema to potentially increase the risk for SCD [192]. Avanesov et al. further showed an association of ECV with SCD [193]. Global ECV predicted SCD superior to LGE size with an area under the curve (AUC) of 0.83.

CMR studies could also report significant strain impairment in HCM [194,195] and furthermore an association to adverse outcome. In a CMR-FT study, GLS, GCS, and GRS were associated with a primary endpoint of all-cause mortality and a secondary combined endpoint of hospital admission related to HF, lethal VA, or cardiovascular death. A review of 14 studies using STE identified an association of impaired LV GLS with a composite endpoint including VA, cardiovascular mortality, and ICD firing.

RCM is a heterogeneous disease that may overlap with HCM and can transform into a DCM phenotype. Various underlying etiologies can lead to RCM such as storage diseases (e.g., iron overload) and infiltrative disorders (above all amyloidosis) [18]. In this context, LGE pattern can provide valuable information regarding the specific subtype [196]. In amyloidosis, SCD is often caused by initial bradycardia and consequent pulseless electrical activity, whereas VA are uncommon; thus, the benefit of ICD implantation is questionable [18,197]. In amyloidosis, LGE is usually found subendocardially both in the ventricle and in the atrium. The proportion of atrial involvement can help differentiate from hypertensive heart disease and DCM [198,199]. Transmural or global LGE extension along the whole LV circumference significantly impairs prognosis [200,201,202]. Further prognostic yield can be achieved by examining T1 values and LV strain. They have been shown to independently predict mortality in AL amyloidosis [198,201,203,204]. It is noteworthy that the discussed studies did not specifically address SCD but overall mortality. Considering that amyloidosis is a multiorgan disease and death may be related to other non-cardiac causes, risk stratification should focus on SCD alone.

Although HCM and RCM represent different entities of myocardial diseases com-pared with NIDCM, the discussed CMR parameters are similarly helpful and applicable in risk stratification of SCD.

## 5. Lack of Evidence in Current Risk Stratification

Studies on NICM are mostly observational, single-center studies that are limited to a small sample size [25,98] and lack uniform endpoints. Due to the overall small number of events, researchers use composite endpoints. However, these are not easily interchangeable, and some endpoints are no substitute for SCD [205].

**Figure 2:**
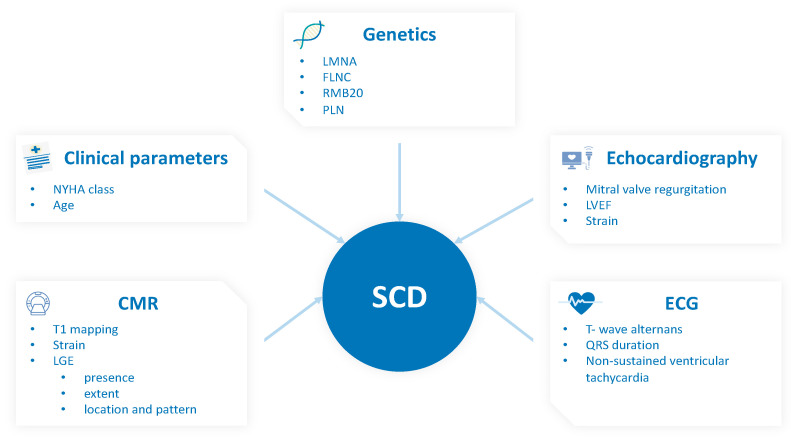
Parameter for risk assessment in patients with non-ischemic cardiomyopathy (NICM). Abbreviations: SCD: sudden cardiac death; CMR: cardiac magnetic resonance; LGE: late gadolinium enhancement; LVEF: left ventricular ejection fraction; NYHA: New York Heart Association.

To summarize, various reasons demand a new, precise risk score for SCD referring exclusively to NICM patients. First, the current clinical recommendations for ICD implantation focus on patients with LVEF ≤ 35%, despite a substantial proportion of patients at risk with higher LVEF and conflicting results of previous ICD trials addressing NICM. Second, NICM represents a heterogenous entity that substantially differs from ICM, needing targeted risk scores for each specific subtype (DCM, ARVC, HCM, etc.). The fact that most studies mix DCM with other NICM patients possibly blurs results and hampers comparability. Finally, genetics and newer imaging modalities such as CMR or STE have not been incorporated in risk stratification tools and guidelines so far, despite increasing importance in clinical routine and mounting evidence supporting their use. Some genetic mutations such as LMNA, RBM20, FLNC, and PLN are clearly linked to arrhythmic phenotypes. CMR techniques including the presence, extent, and distribution of LGE, mapping, and strain have also been shown to add value to risk stratification. For a summary of all discussed risk parameters, see Figure 2.

## 6. Future Perspectives

Investigations using CMR for risk stratification of SCD still lack larger prospective randomized trials [174]. New insights are hoped to be gained from the still ongoing CMR-Guide Trial ([206] (NCT01918215)) including patients with mild to moderate reduced EF and the presence of LGE who are randomized to loop recorder or primary preventive ICD implantation. All patients are on optimal medical HF therapy and followed for 4 years. The primary endpoint is defined as time to SCD or hemodynamically significant VA (VF or VT). Results are expected for 2024. The authors hypothesize that in patients with mild to moderate EF reduction, a CMR-guided strategy for ICD implantation based on the presence of LGE is superior to the current standard care. Another recently started multi-center, prospective, randomized, and controlled study, the CMR-ICD-DZHK23 trial (NCT04558723), investigates NIDCM patients with an LVEF < 35% and myocardial fibrosis detected by CMR. Patients are either randomized to ICD implantation or optimal medical HF therapy including CRT-P treatment. The goal of the study is to find out whether ICD implantation reduces overall mortality in NIDCM patients with an LVEF < 35% and with myocardial fibrosis on CMR compared to optimal medical treatment alone.

All in all, to fill current knowledge gaps for risk stratification, future studies should focus only on patients with NICM and assess patients irrespective of LVEF. Such trials should evaluate on the one hand whether LGE can help to discriminate patients with ICD indication for primary prevention according to current guidelines who nevertheless may not benefit in terms of survival. On the other hand, studies should identify patients without ICD indication based on current guidelines, who are yet at increased risk for SCD and who might therefore benefit from ICD implantation. Finally, as no single risk factor to date has the discriminant power for safely identifying patients at risk for SCD, clinical, genetic, and imaging parameters should be considered simultaneously.

## Figures and Tables

**Table 1:** Overview of CMR parameters and their limitations in risk assessment for SCD.

Parameter	Key Points	Limitation
LGE	Visualization of myocardial fibrosis as substrate for VA Presence as idependent predictor for VA and SCD Contradictory findings concerning role of extent, localization, and pattern	Contraindications to contrast agent use Different methods to define presence of scar Different methods to quantify scar extent
T1 mapping/ECV	Marker of diffuse fibrosis Higher native T1 values are associated with arrhythmic endpoints Applicable independent of renal function	Susceptibility to confounding variables during acquisition, e.g., gadolinium dose, rate of injection Lack of standardization of mapping techniques Lack of standardization of post-processing techniques Vendor-dependent cut-off values Overlap with T1 values of normal myocardium in early disease stages of NICM
Strain imaging	Parameter for myocardial deformation and function Impaired strain asssociated with adverse outcome	Lack of validation for some strain assessment methods Method and software specific cut-off values Lack of reliability concerning some strain parameters, e.g., radial and segmental strain Lack of larger studies Lack of studies focusing solely on arrhythmic endpoints
